# A phase II study of cabozantinib and pembrolizumab in advanced gastric/gastroesophageal adenocarcinomas resistant or refractory to immune checkpoint inhibitors

**DOI:** 10.1093/oncolo/oyae117

**Published:** 2024-06-01

**Authors:** Farshid Dayyani, Joseph Chao, Fa-Chyi Lee, Thomas H Taylor, Kristen Neumann, May T Cho

**Affiliations:** Division of Hematology/Oncology, Department of Medicine, University of California Irvine, Orange, CA 92868, United States; Department of Medical Oncology and Therapeutics Research, City of Hope, CA 91010, United States; Division of Hematology/Oncology, Department of Medicine, University of California Irvine, Orange, CA 92868, United States; Department of Epidemiology and Biostatistics, University of California Irvine, Irvine, CA 92617, United States; Division of Hematology/Oncology, Department of Medicine, University of California Irvine, Orange, CA 92868, United States; Division of Hematology/Oncology, Department of Medicine, University of California Irvine, Orange, CA 92868, United States

**Keywords:** gastroesophageal cancer, immune checkpoint inhibitor, ICI, TKI

## Abstract

**Background:**

Most patients with metastatic gastroesophageal adenocarcinoma (mGEA) progress on immune checkpoint inhibitors (ICIs). Novel approaches to overcome resistance to ICI in mGEA are needed. Cabozantinib is a multi-tyrosine kinase inhibitor thought to enhance the immunomodulatory effects of ICI. This study evaluated the combination of cabozantinib and pembrolizumab in ICI refractory or resistant mGEA.

**Methods:**

Investigator-initiated, single‐arm, single institution, and phase II study in patients with mGEA. Patients had progressed on ICI and/or had PD-L1 CPS score ≤10%. Cabozantinib dose was 40 mg p.o. daily on days 1-21 of a 21‐day cycle, with pembrolizumab 200 mg i.v. on day 1. The primary endpoint was progression-free survival at 6 months (PFS-6).

**Results:**

Twenty-seven patients were enrolled. Median age 58 years (24-87), female (*n* = 14), ECOG 0/1 = 13/14, GC/GEJ = 16/11, and non-Hispanic White/Hispanic/Asian = 12/8/7. The primary endpoint was met. After a median follow-up of 31.4 months (range 3.3-42.5), PFS-6 was 22.2% (95% CI 9.0-39.0). The median PFS and OS are 2.3 months (95% CI 1.7-4.1) and 5.5 months (3.1-14.0), respectively. The most common mutations were TP53 (78.3%) and CDH1/PIK3CA/CTNNB1 (17.4% each). The most common grade (G) treatment-related adverse events (TRAE) were diarrhea (25.9%), fatigue (18.5%), hypertension, and muscle cramps (14.8% each). G3-4 TRAE were seen in *n* = 3 patients (hypertension, thromboembolic event, esophageal perforation; each *n* = 1). No G5 was observed.

**Conclusions:**

The addition of cabozantinib to pembrolizumab shows clinical benefit in ICI-resistant or refractory mGEA with a tolerable safety profile. (ClinicalTrials.gov Identifier: NCT04164979. IRB Approved: UCI 18-124, University of California Irvine IRB#20195426.)

## Teaser

Novel approaches to overcome resistance to immune checkpoint inhibitors (ICIs) are needed. This study evaluated the combination of cabozantinib and pembrolizumab in ICI refractory or resistant metastatic gastroesophageal adenocarcinoma.

**Lessons learned**

- Most patients with gastroesophageal adenocarcinomas progress on immune checkpoint inhibitors.
- Cabozantinib is thought to enhance the immunomodulatory effects of immune checkpoint inhibitors.
- Cabozantinib plus pembrolizumab is active in patients refractory to immune checkpoint inhibitors.
- Progression-free survival at 6 months was 22.2%, more than 4-fold higher than prior studies.
- The adverse event profile was as expected and did not reveal any new safety signals.

## Discussion

Gastric cancer is the third leading cause of cancer mortality and the fifth most common malignancy worldwide.^1^ In patients with PD-L1-positive tumors (CPS score ≥ 5%), the addition of the immune checkpoint inhibitor (ICI) to chemotherapy is recommended.^2-5^ In the Keynote-59 trial, pembrolizumab produced an ORR of 11.6% in 3L + mGEA, although the response rate was higher (15.5% vs 6.4%) in patients with PD-L1 positive compared to PD-L1 negative tumors (defined as CPS score ≥ 1%).^6^ While the responses were durable, the 6-month PFS (6-PFS) was only 14.1% and the median progression-free survival (PFS) was 2.0 months. These findings highlight the remaining unmet need for most patients who either are refractory (low PD-L1 CPS score) or develop disease progression following treatment with ICI.

Cabozantinib is a potent inhibitor of 3 principal targets: VEGF-R, cMET, and Axl.^7^ Cabozantinib has been safely combined with PD-1 and PD-L1 inhibitors in solid tumors.^8,9^ A recently reported study examined the efficacy of cabozantinib in patients with advanced renal cell carcinoma who progressed on ICI.^10^

We hypothesized that cabozantinib, based on preclinical and clinical observations so far, might contribute to overcoming primary or secondary resistance to ICI in mGEA. Thus, we conducted a phase II study to estimate the efficacy of cabozantinib and pembrolizumab in this cohort.

The primary endpoint was met with 6 patients (22.2%) having PFS > 6 months. Two patients (7.4%) remained on study for the entire duration of treatment, ie, 24 months. In all 27 patients, the rate of disease control was 17 of 27, ie, 62.3%. In an exploratory analysis, 5 of 7 patients (71.4%) in the top quartile for PFS had tumor alterations in the cMET-PI3K-AKT pathway vs 2 of 7 patients in the bottom quartile (28.6%). A total of 5 grade 3/4 TRAE were recorded (esophageal perforation, *n* = 2; hypertension, stroke, and thromboembolic event, each *n* = 1). The most common TRAEs were diarrhea (*n* = 9), fatigue (*n* = 6), dysgeusia, and hypertension (each *n* = 5). There were no grade 5 TRAEs.

**Figure 1.**
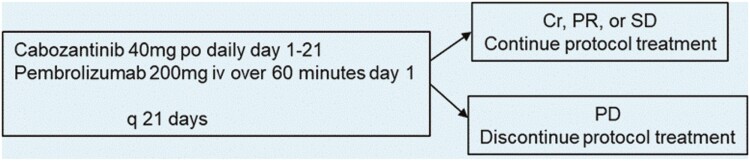
Protocol treatment.

**Figure 2.**
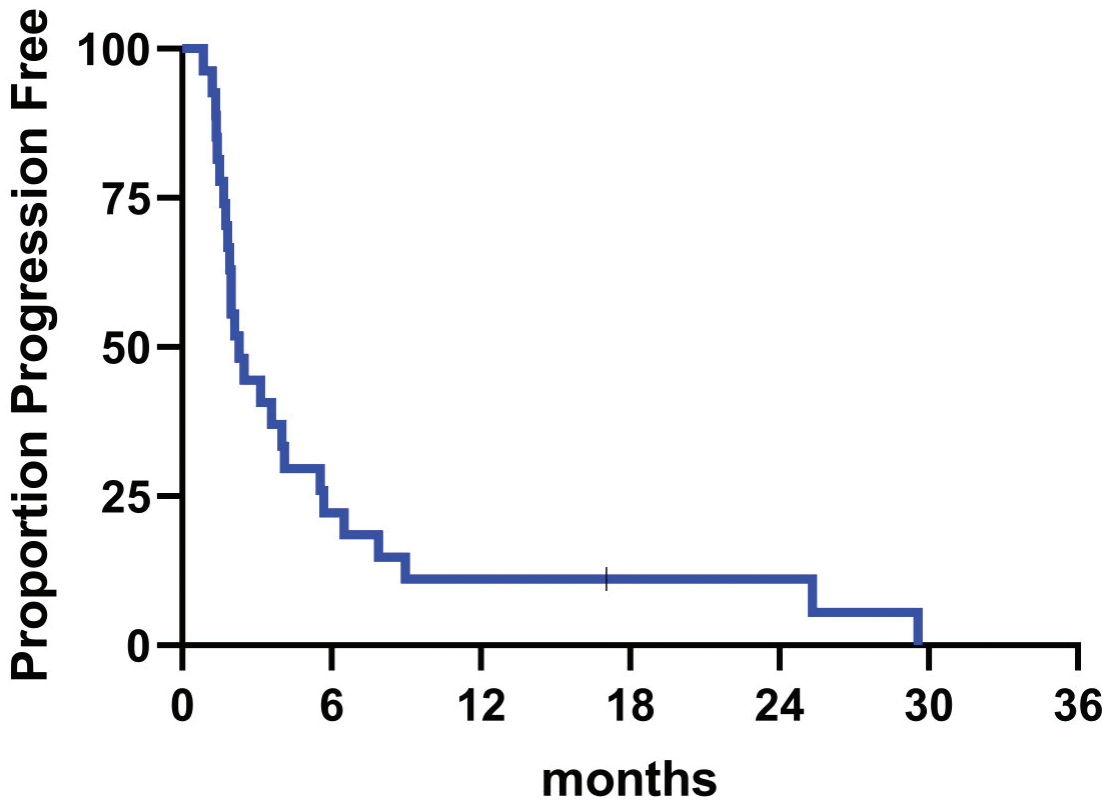
Proportion progression free.

Cabozantinib plus pembrolizumab appears to be effective in mGEA resistant or refractory to ICI-based on CPS score (Figures 1 and 2).

## Trial Information

**Table:** 

Disease	Gastric or esophageal adenocarcinoma
Stage of disease/treatment	Locally advanced, recurrent, or metastatic and not amenable to curative intent surgery
Prior therapy	At least one line of therapy including a fluoropyrimidine and platinum regimen for advanced disease. Patients with unknown tumor PD-L1 CPS score, those with CPS ≥ 10%, and patients with MSI-High or MMR deficient tumors were required to have progression on at least one line of prior therapy with an ICI containing regimen.
Type of study	Single institution, single-arm, open-label, phase II clinical trial
Primary endpoint	Six months progression-free survival (PFS-6)
Secondary endpoints	Rates of drug-related grade 3-5 adverse events; objective response rate by RECIST v1.1; median progression-free (mPFS); median overall survival (mOS)
Additional details of endpoints or study design	PFS was defined as the time from the start of treatment to progression or death, and OS was defined as the time from start of treatment to death

## Drug Information

**Table:** 

Drug 1: generic/working name	Cabozantinib
Company name	Exelixis
Drug type	Biologic
Drug class	Tyrosine kinase inhibitors
Dose	40 mg
Route	Oral
Schedule of administration	Once daily
Drug 2: generic/working name	Pembrolizumab
Company name	Merck
Drug type	Anti-PD1 mAb
Drug class	200 mg
Route	i.v.
Schedule of administration	Every 21 days

## Patient Characteristics

**Table:** 

Number of patients, male	13
Number of patients, female	14
Stage	III (*n* = 9), IV (*n* = 18)
Age: median (range)	58 (24-87) years
Number of prior systemic therapies: median (range)	2 (1-5)
Performance status: ECOG	0: 13 1: 14 2: 0 3: 0 4: 0
Cancer types or histologic subtypes	Intestinal type, 5; diffuse type, 20; mixed type, 2

## Primary Assessment Method

**Table:** 

Number of patients screened	28
Number of patients enrolled	27
Number of patients evaluable for toxicity	27
Number of patients evaluated for efficacy	27
Evaluation method	RECIST 1.1
Response assessment, PR	1 (7.7%)
Response assessment, SD	7 (41.2%)
Response assessment, PD	5 (38.5%)
Median duration assessments, PFS	2.3 months (95% CI: 1.7-4.1)
Median duration assessments, PFS-6	22.2%
Median duration assessments, OS	5.5 months (95% CI: 3.1-14.0)

## Outcome notes

**Figure 3.**
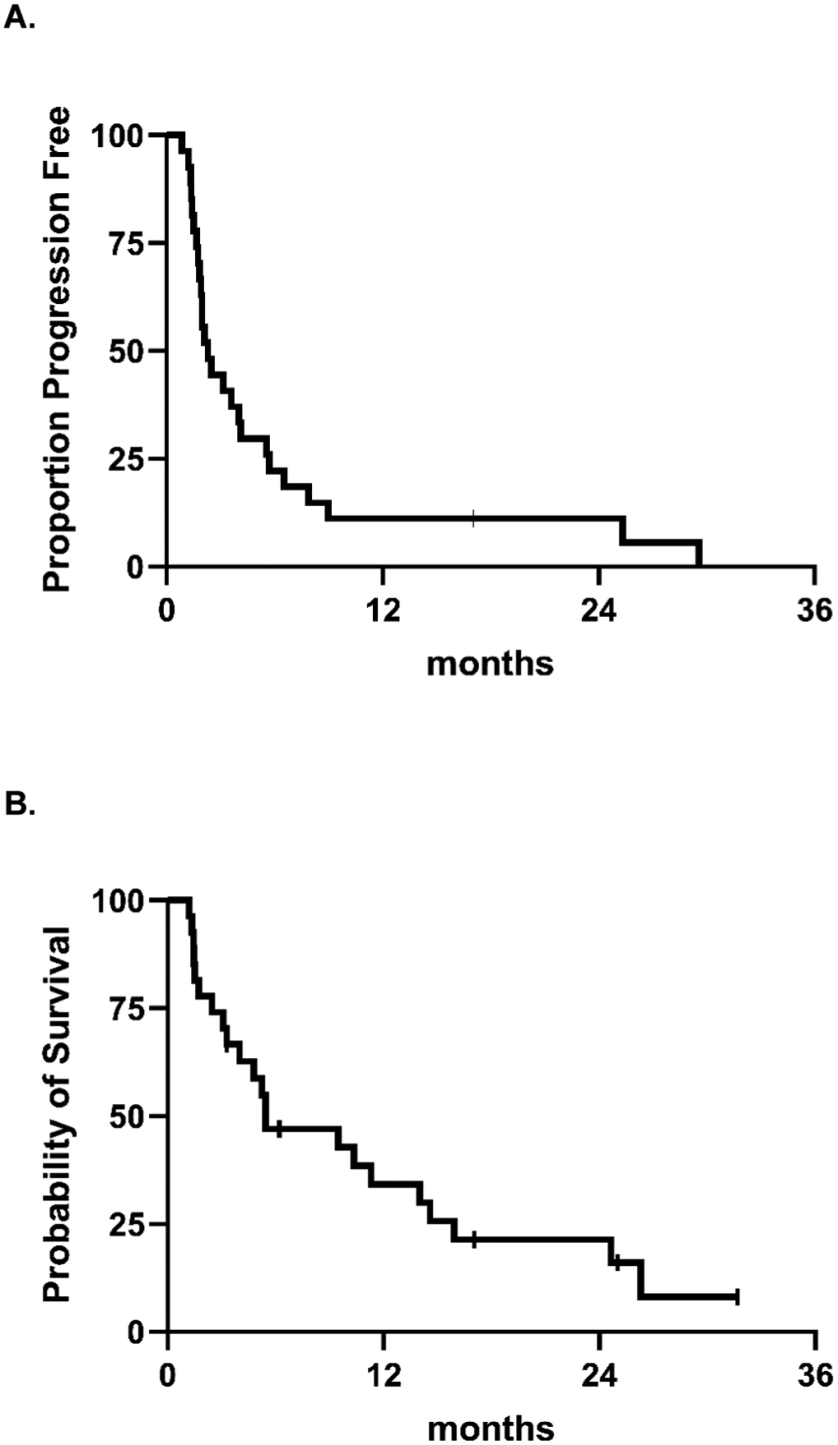
Progression-free survival (A) and overall survival (B).

**Figure 4.**
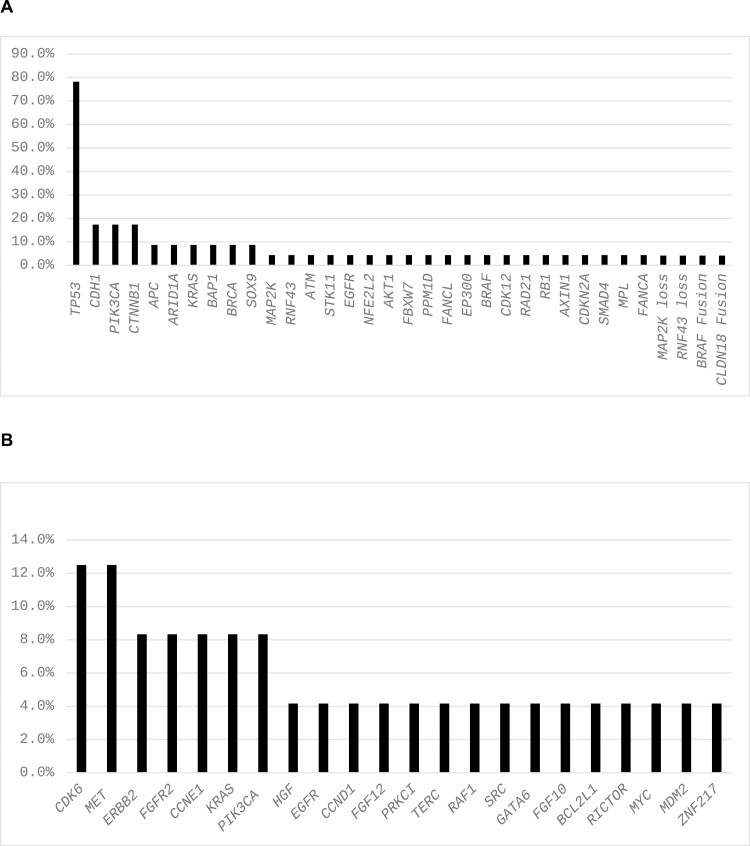
Molecular alterations (A) point mutations/fusions and (B) amplifications.

The primary endpoint was met with 6 patients (22.2%) having PFS > 6 months. Two patients (7.4%) remained on study for the entire duration of treatment, ie, 24 months. After a median of 31.4 months (range 3.3-42.5), the median PFS and OS were 2.3 months (95% CI 1.7-4.1) and 5.5 months (95% CI 3.1-14.0), respectively. In 13 patients with measurable disease, best objective response by RECIST v1.1 was partial response in 1 patient (7.7%), stable disease in 7 patients (41.2%), and progressive disease in 5 (38.5%) patients. Among the remaining 14 patients with evaluable but not RECIST measurable disease at baseline, 9 showed no progression at the first imaging timepoint and continued treatment beyond 8 weeks. The other 5 patients progressed before the first on-treatment imaging. Hence, in all 27 patients, the rate of disease control was 17 of 27, ie, 62.3% (Figures 3 and 4, Table 1).

**Table 1.** Patient baseline characteristics (*N* = 27).

Characteristics	*n* (%)
Age, years	
Median	58 years
Range	24-87 years
Gender, *N* (%)	
Male	13 (48)
Female	14 (52)
Ethnicity, *N* (%)	
Caucasian	12 (44)
Hispanic	8 (30)
Asian	7 (26)
ECOG performance status, *N* (%)	
0	13 (48)
1	14 (52)
Tumor location, *N* (%)	
Stomach	16 (59)
Gastroesophageal junction	11 (41)
Stage at diagnosis, *N* (%)	
Locoregional	9 (33)
Metastatic	18 (67)
Histologic type, *N* (%)	
Intestinal	5 (19)
Diffuse	20 (74)
Mixed	2 (7)
Mismatch repair, *N* (%)	
Proficient	27 (100)
Her-2 positive, *N* (%)	
Yes	3 (11)
No	24 (89)
PD-L1 CPS score, *N* (%)	
≥ 1%	23 (85)
≥ 5%	15 (56)
≥ 10%	12 (44)
Prior ICI, *N* (%)	
Yes	19 (70)
No	8 (30)
Site of metastases, *N* (%)	
Liver	4 (15)
Lung	1 (4)
Lymph nodes	17 (63)
Peritoneum	15 (56)
Bone	4 (15)
Other	8 (30)
Number of metastases, *N* (%)	
1	11 (41)
2	11 (41)
≥3	5 (19)
Number of prior systemic treatments for metastatic disease, *N* (%)	
1	11 (60)
2	8 (30)
≥3	8 (30)
Type of prior systemic treatments, *N* (%)	
Fluoropyrimidine	27 (100)
Platinum	27 (100)
Irinotecan	4 (15)
Taxane	15 (56)
Anti-Her-2	4 (15)
Other	14 (52)
Prior surgery, *N* (%)	
Yes	9 (33)
No	18 (67)
Prior radiation, *N* (%)	
Yes	7 (26)
No	20 (74)

## Results of pharmacodynamic analysis

All patients who started treatment were evaluated for adverse event (AE) assessment. Table 2 shows treatment-related adverse events (TRAE) occurring in ≥5% of patients (ie, more than 1 patient). All patients reported at least one TRAE (total AEs reported *n* = 57). A total of 5 grade 3/4 TRAE were recorded (esophageal perforation, *n* = 2; hypertension, stroke, and thromboembolic event, each *n* = 1). The most common TRAEs were diarrhea (*n* = 9), fatigue (*n* = 6), dysgeusia, and hypertension (each *n* = 5). There were no grade 5 TRAEs. Dose delays occurred in 11 patients for cabozantinib and in 1 patient for pembrolizumab. Dose reductions for cabozantinib occurred in 9 and 2 patients to dose levels 1 and 2, respectively (ie, 20 mg daily and 20 mg every other day).

**Table 2.** Treatment-related adverse events (≥ 5% frequency).

TRAE	G1	G2	G3	G4
Diarrhea	8	1	0	0
Fatigue	3	3	0	0
Dysgeusia	4	1	0	0
Hypertension	2	2	1	0
Muscle cramps	0	4	0	0
AST elevation	3	0	0	0
Bruising	3	0	0	0
Dry skin	3	0	0	0
Mucositis	3	0	0	0
PPES	1	2	0	0
Thromboembolic event	0	1	1	0
Esophageal perforation	0	0	0	2

Abbreviations: AST, aspartate aminotransferase; PPES, palmar-plantar erythrodysesthesia syndrome; TRAE, treatment-related adverse events.

## Assessment, Analysis, and Discussion

**Table:** 

Completion	Study completed
Investigator’s assessment	Active and should be pursued further

Based on the observations of the Keynote 059 study^11^ and others which showed durable responses in a minority of mGEA patients treated with single agent pembrolizumab in the salvage setting, we hypothesized that ICI would be moved to the front line setting over time but also realized that only a minority of patients would have long-term benefit and therefore strategies to overcome resistance to ICI would be needed. Since then, multiple positive phase III trials^12-14^ have changed the treatment landscape in first-line mGEA and ICI are now being used in combination with chemotherapy for CPS PD-L1-positive tumors. However, even in CPS > 5% tumors, approximately 2/3 of the patients have progressed by 12 months and only 1 in 8 remain free of progression by 36 months.^14^

We show the combination of cabozantinib with pembrolizumab is feasible and active in a real-life population of primary resistant or ICI refractory mGEA. In this single arm study, we sought to aim for a large effect size (ie, more than 4-fold increase in the PFS-6 expected in previously published data).

With these considerations in mind, we specifically chose PFS-6 as the primary endpoint of this study. This allowed us to maximize the number of eligible patients (since not based on objective response rate) and minimize sample size (since we aimed for a relatively large effect size). Given the geographic location of our center (California) with a high proportion of patients with Asian and Hispanic ethnicity (56%), we had a relatively high proportion of patients with associated aggressive histology (poorly differentiated and or signet cell histology).^15^

We chose the definition of ICI refractory based on the secondary analyses of the single-agent pembrolizumab arms of the keynote 059 and 061 studies,^6,16^ which showed the greatest benefit of single-agent anti-PD-1 inhibition in mGEA in patients with a CPS score greater than 10%. Hence, we allowed enrollment of patients who were ICI naïve but whose tumors had a CPS score of less than 10%. Even though our enrollment started in 2018, ie, before the approval of ICI and first-line treatment of mGEA, 2/3 of our patients had prior exposure to ICI. Additionally, it is important to note that among the remainder of 9 patients who were ICI naïve, all but one, ie, 88.9% had tumors with CPS scores less than 5%. Hence, the observed PFS-6 of 22.2% in this population appears to be a meaningful improvement over the expected PFS-6 of around 5%.

Sixty percent of our patients had received at least 2 lines of therapy (ie, 3L plus setting). Of the remaining 40% (*n* = 11) had either received a triplet regimen in first line with a taxane or an anti-PD-1 antibody (*n* = 9) or had neuropathy which would have precluded treatment with a taxane (*n* = 2).

Recently a phase Ib basket study evaluated the combination of cabozantinib plus durvalumab, an anti-PD-L1 monoclonal antibody, in patients with colorectal carcinoma, hepatocellular carcinoma and mGEA. Ten patients with mGEA were enrolled. Three of them had an objective response. Important differences to our study include the fact that all the patients in this trial where checkpoint inhibitor naïve, almost 90% were White, and data regarding PD-L1 CPS score of the patients with mGEA were not presented.^17^

Somatic mutational analysis was available for the majority (24 of 27) of patients in our study. While not prespecified and somewhat limited due to retrospective nature of the analysis and relatively small sample size, we made some notable observations. Firstly, TP53 mutations were by far the most frequently detected mutations in our patient cohort (78.3%). A recent report of over 3400 patients with gastric and gastroesophageal junction (GEJ) adenocarcinomas showed a higher prevalence of TP53 mutations in GEJ vs gastric cancers (61.6% vs 81.4%).^18^ Of note, 3 of 27 patients had an MET amplification, and 2 of those patients had a PFS in the top quartile. Additionally, 3 of 7 patients in the top quartile for PFS had mutations in the MET signaling pathway (ie, PIK3CA and AKT 1). While interesting, the retrospective and exploratory nature of these observations, together with a relatively small sample size, make any definite conclusion regarding predictive biomarkers for this regimen based on our study challenging. These findings would have to be prospectively validated in a future prospective and larger follow-up study.

However, while the role of MET signaling in the progression and prognosis of gastric cancer has been well documented,^19-21^ achieving therapeutic success via specific anti-MET targeted therapies have thus far proven to be challenging in the treatment for gastric cancers.^22^ Hence, rather than enrolling patients based on MET amplification, we selected cabozantinib in our study mainly because of the presumed immunomodulatory activity of the compound,^23^ which includes inhibition of MET, Axl, and VEGFR but also other targets present in an immune suppressive tumor microenvironment such as the TAM kinase family.^24-26^

The single arm, single center design of the trial is a potential limitation of the study. However, the diverse patient population included likely makes the results applicable to a wide range of geographic locations. The non-randomized design did not allow for the assessment of the activity of cabozantinib alone. An earlier phase II discontinuation trial evaluated single-agent cabozantinib in solid tumors and did not demonstrate the significant clinical activity of single-agent cabozantinib in the mGEA cohort.^27^ Hence our data appear to indicate that the activity noted in this ICI refractory setting is likely not attributed to cabozantinib alone.

In summary, cabozantinib combined with pembrolizumab appears to be a feasible treatment option for patients with mGEA who have progressed on prior ICI containing regimens or who are not eligible for ICI-based on a low/negative PD-L1 CPS score.

## Data Availability

The data underlying this article will be shared on reasonable request to the corresponding author.
